# Construction and validation of a dimensional scale exploring mood disorders: MAThyS (Multidimensional Assessment of Thymic States)

**DOI:** 10.1186/1471-244X-8-82

**Published:** 2008-09-19

**Authors:** Chantal Henry, Katia M'Bailara, Flavie Mathieu, Rollon Poinsot, Bruno Falissard

**Affiliations:** 1 AP-HP, Henri Mondor-Albert Chenevier Hospitals, Department of Psychiatry, Creteil, F-94000, France; 2 INSERM, U 841, IMRB, dept of Genetics, Psychiatry Genetics, Creteil, F-94000, France; 3 Université Victor Segalen, EA 4139, F-33000, France; 4 Université Paris 8, Saint-Denis, F-93526, France; 5 Inserm, U669, Paris, F-75679, France; 6 Univ Paris-Sud and Univ Paris Descartes, UMR-S0669, Paris, F-75679, France; 7 AP-HP, Hôpital Paul Brousse, département de santé publique, Villejuif, F-94804, France

## Abstract

**Background:**

The boundaries between mood states in bipolar disorders are not clear when they are associated with mixed characteristics. This leads to some confusion to define appropriate therapeutic strategies. A dimensional approach might help to better define bipolar moods states and more specifically those with mixed features.

Therefore, we proposed a new tool based on a dimensional approach, built with a priori five sub-scales and focus on emotional reactivity rather than exclusively on mood tonality. This study was designed to validate this MAThyS Scale (Multidimensional Assessment of Thymic States).

**Methods:**

One hundred and ninety six subjects were included: 44 controls and 152 bipolar patients in various states: euthymic, manic or depressed. The MAThyS is a visual analogic scale consisting of 20 items. These items corresponded to five quantitative dimensions ranging from inhibition to excitation: emotional reactivity, thought processes, psychomotor function, motivation and sensory perception. They were selected as they represent clinically relevant quantitative traits.

**Results:**

Confirmatory analyses demonstrated a good validity for this scale, and a good internal consistency (Cronbach's alpha coefficient = 0.95). The MathyS scale is moderately correlated of both the MADRS scale (depressive score; r = -0.45) and the MAS scale (manic score; r = 0.56).

When considering the Kaiser-Guttman rule and the scree plot, our model of 5 factors seems to be valid. The four first factors have an eigenvalue greater than 1.0 and the eigenvalue of the factor five is 0.97. In the scree plot, the "elbow", or the point at which the curve bends, indicates 5 factors to extract. This 5 factors structure explains 68 per cent of variance.

**Conclusion:**

The characterisation of bipolar mood states based on a global score assessing inhibition/activation process (total score of the MATHyS) associated with descriptive analysis on sub-scores such as emotional reactivity (rather than the classical opposition euphoria/sadness) can be useful to better understand the broad spectrum of mixed states.

## Background

The heterogeneity of mood episodes is a crucial issue especially in bipolar disorders and leads often to some confusion in diagnostic and therapeutic strategies. Apart from the classical syndromes characterizing euphoric mania and melancholic depression, recent literature has pointed to alternative mood states associating both manic and depressive symptoms. This resulted in the definition of various syndromes including mixed states, dysphoric mania [1], agitated depression [2], depressive mixed state [3,4] and more recently mixed hypomania [5,6]. As a consequence, this leads to question the best therapeutic strategies [5]. As the boundaries between the various states associating both depressive and manic symptoms have yet to be clarified, there is a need to explore whether dimensional approaches could help to refine their definitions [1,7,8].

With a very modern point of view, Kraepelin [9] defined mood states as originating from the excitement or inhibition of the three domains of the psyche: cognitive processes (train of thought rather than its contents), mood, and volition (expressed in psychomotor activity). We have extended this notion, by replacing mood tone (euphoria vs. sadness) by emotional reactivity (hyporeactive vs. hyperreactive), which is closer to the concept of dimensions, and because it may be considered as a quantitative symptom. An emotion is characterized not only by its tone (pleasant/unpleasant), but also by its intensity or reactivity. The understanding of the different processes underlying emotion is still limited and there is not a generally accepted theoretical framework. However, it seems that in presence of an emotive stimulus there are: 1) the identification of the emotion and significance of the stimulus; 2) the production of a specific affective state in response to the stimulus (including physiologic arousal); 3) the regulation of the affective state and emotional behavior, which may involve an inhibition or modulation of the two preliminary processes in order to produce an affective state and a behavior appropriate to the context [10]. With emotional reactivity we explore process 2 and 3.

In concrete terms, all depressive states are characterized by sadness, so affective mood tone cannot distinguish between different types of depression. However, the quantitative component of emotions can provide a useful discriminatory element. Indeed, emotional reactivity can be inhibited, leading to a loss of pleasure or anhedonia, which, in its most complete expression, results in true emotional anesthesia. Kraepelin [9] described certain depressive patients as insensitive even to bad news. Such emotional anesthesia was also described by Goodwin and Jamison [11] in traditional slowed down depressions. However, some bipolar depressions with atypical features are not characterized by an emotional hyporeactivity. Conversely, manic and mixed states were found to be better characterized by emotional hyperreactivity than by affective tone, which is very variable [12]. Emotional hyperreactivity implies that emotions are felt with a greater intensity than usual and that they vary according to environmental stimulations. Thus, emotional reactivity might be useful to discriminate states with mixed features.

Based on these concepts we developed a tool called MAThyS (Multidimensional Assessment of Thymic States) (see Additional file 1). This is a visual analogic scale based on a dimensional approach aiming to discriminate between different sub-populations among patients suffering from bipolar disorders. The instrument is designed as a multi-dimensional assisted self-administered questionnaire comprising 20 items relating to individual states as perceived by patients for the preceding week. Each item is set out as a continuous measure in the form of a visual analogic scale of 10 cm on which the subject is asked to make a mark to indicate where he/she is positioned between the two predefined extreme propositions.

The scale is developed using five *a priori *dimensions which can fluctuate from inhibition to excitation to explore mood episodes and represent quantitative dimensions (see annex). The five dimensions are: emotional reactivity, cognition speed, psychomotor function, motivation and sensory perception. Because the dimensions assess inhibitory or activation processes, they can be applied to manic or depressive states as well as to states presenting with an admixture of both. Emotional reactivity may be considered as a new component in comparison of current mood scales and seems appropriate to define mixed states.

The objective of MAThyS is to define bipolar mood states as a function of an inhibition/activation process using a dimensional approach. According to this concept, mood is defined using emotional reactivity rather than tonality of affects. This approach can help to order bipolar mood states on a continuum and to define a spectrum of mixed states.

The aim of the study is to present elements of validation of the MAThyS Scale (Multidimensional Assessment of Thymic States), with an account of the reliability and construct validity of this assisted self-administered questionnaire.

## Methods

### Subjects

The first group included control subjects, without bipolar disorder, recruited by means of an advertisement (for example in shopping areas and in sportive associations). Euthymic bipolar outpatients were recruited from consultations, and bipolar patients presenting with a depressive, manic, hypomanic or mixed episodes were recruited from consecutive admissions as inpatient in a unit of general psychiatry and as outpatients in a consultation for bipolar disorders corresponding to a specific geographic area and thus very representative of a general population of bipolar patients (Charles Perrens Hospital, Bordeaux, France).

Patients and controls were interviewed by a trained psychologist using the section of mood disorders of the French version of the Diagnosis Interview for Genetic Studies [13] providing DSM-IV diagnosis [14]. Subjects with current alcohol or substance misuse were excluded. For inclusion in the group of euthymic bipolar patients, subjects did not, at the time of the evaluation, fulfill the criteria for a major depressive episode or a manic, mixed or hypomanic episode, according to DSM-IV criteria. Euthymia was confirmed by a general clinical evaluation, carried out by the treating psychiatrist, and by low scores on depressive and manic scales (MADRS ≤ 12 and MAS ≤ 4) [15,16]. Patients were included after giving informed consent and did not receive any financial compensation (controls and bipolar patients). The study was approved by the Ethics Committee.

### MAThyS scale

#### Administration of the scale

MAThyS is a self-completed questionnaire filled in with assistance at least for the first completion. The evaluation concerns mood during the last week. Because it is a visual analogic scale, the patient must choose between the two proposed statements for each item and then indicate, with a vertical line, his or her state. When the patient is in is basal state, the vertical line should be marked in the centre of the horizontal line between the two proposed statements. By contrast, if the patient's mood fluctuates, he or she should decide which of the two proposed statements best describes his or her current state. The vertical line should then be marked between the centre and the selected statement, the precise position with respect to the extremity of the horizontal line depending on the extent to which the patient identifies with the statement. The time to fill in the MAThyS is about 10 minutes.

#### Scoring

Score is determined line-by-line and varies from 0 to 10 for each line. A score of 0 corresponds to inhibition of the state evaluated by the item. A score of 5 indicates no change from the patient's usual state and a score of 10 corresponds to excitation for the evaluated state. An overall score between 0 and 200 is thus obtained. This scale is not devoted to make a diagnosis of mood state but allow to determine a general level of inhibition/activation processes (lower scores indicate general inhibition and higher scores indicate general excitation). A more descriptive approach can be done by analysing the sub-scores for each dimension (table 1). The measure is the number of centimetres from the left hand anchor. Items measured from 0 to 10 are: 1; 2; 3; 4; 11; 12; 13; 14; 15; 16; 19; 20 and items measured from 10 to 0 are: 5; 6; 7; 8; 9; 10; 17; 18. A verbatim and a guidebook are available and can be provided by the corresponding author.

**Table 1:** The hypothetical structure of the MAThyS: description of the five a priori dimensions of the MATHYS (emotion, cognition, psychomotor function, motivation, sensory perception) with the corresponding items.

Dimension	Dimension name	Items numbers	Continuum
EM	EMOTION	3, 7, 10, 18	Hypo-reactivity/Hyper-reactivity
CO	COGNITION	5, 9, 12, 14	Retardation/Acceleration
MO	PSYCHOMOTOR FUNCTION	2, 11, 19	Retardation/Acceleration
VO	MOTIVATION	4, 15, 16, 17	Decrease/Increase
SE	SENSORY PERCEPTION	1, 6, 8, 13, 20	Decrease/Increase

### Statistical analyses

First a descriptive analysis of items explored their distribution (missing data, normality, scatter of responses, floor and ceiling effects) and redundancy (estimation of Pearson's correlation coefficient between items two by two, with a threshold at 0.70).

The internal consistency of the scale was estimated with the Cronbach Alpha coefficients. Elements of external validity were assessed by comparing the MAThyS to the MADRS and the MAS.

The screeplot of the correlation matrix of the 20 items was drawn, the number of dimensions of the scale was appreciated with application of Kaiser's criterion (eigenvalues > 1) and Horn procedure (random simulations of data sets). To defined the MAThyS structure, we have performed a classical exploratory factor analyses using the most commonly used orthogonal rotation procedure (Varimax). The number factors to be extracted was fixed at 5, as proposed in the theorical model.

All these analysis were conducted using SAS9.1 package (SAS Institute).

## Results

### Sample characteristics

The sample of 196 subjects was composed of 61 (31.12%) men and 135 (68.88%) women, with a mean age at interview of 38.36 (± 12.75) years.

Seventy one (36.22%) patients had always been single, 85 (43.37%) patients were married or in cohabitation, and 39 (19.9%) were separated or widowed. Most patients presented type I bipolar disorder 92 (60.53%). The socio-demographic data and the repartition in each group is summarized in table 2.

**Table 2:** Socio-demographic characteristics of the sample

	**Total**	**Control**	**Euthymic**	**MDE**	**MDE+Ma**	**Manic**
N	**N = 196**	**N = 44**	**N = 43**	**N = 30**	**N = 28**	**N = 51**

Percentage		**22.4%**	**21.9%**	**15.3%**	**14.3%**	**26.1%**

**Age**						
Mean (+/- sd)	38.4 (+/- 12.75)	35.4 (+/- 12.93)	39.6 (+/- 12.90)	38.9 (+/- 11.89)	42.4 (+/- 10.20)	37.3 (+/- 13.87)
Min-Max	14–78	22–67	17–78	20–61	19–60	14–65
						
**Gender**						
Men (%)	61 (31.1%)	17 (38.6%)	14 (32.6%)	7 (23.3%)	4 (14.3%)	19 (37.2%)
Women (%)	135 (68.9%)	27 (61.4%)	29 (67.4%)	23 (76.7%)	24 (85.7%)	32 (62.7%)
						
**Marital Status**						
Single (%)	71 (36.2%)	19 (43.2%)	18 (41.9%)	7 (23.3%)	6 (21.4%)	21 (41.2%)
Married (%)	85 (43.4%)	23 (52.3)	18 (41.9%)	12 (40%)	15 (53.6%)	17 (33.3%)
Separed/widowed (%)	39 (19.9%)	2 (4.5)	7 (16.3%)	10 (33.3%)	7 (25%)	13 (25.5%)
						
**Bipolar type**						
Type I (%)	92 (60.5%)	-	24 (55.8%)	15 (50%)	8 (28.6%)	45 (88.2%)
Type II (%)	60 (39.5%)	-	19 (44.2%)	15 (50%)	20 (71.4%)	6 (11.8%)

MDE: major depressive episode

MDE+Ma: major depressive episode associated with manic symptoms

Manic: manic, hypomanic and mixed patients

### Acceptability

Only one subject in the group of depressive patients and eight in the "manic" group (including hypomanic and mixed states) did not complete the questionnaire, which means that the participation rate is satisfactory for both these groups at 96.4% and 86.3%. The total number of questionnaires filled in is equal to 187.

All respondents completed all items in the scale (no missing data), which is in favour of good acceptability of the instrument, and suggests it was well understood and easy to complete.

### Item analysis

This analysis was conducted on the whole sample population. In average, the responses were towards the centre of the visual analogic scales, the mean for each item being around 5 (range 4.43 to 6.33). This observation could be linked to the instructions provided, which indicate that "the centre of the line presents your usual state". The dispersion of responses is also similar from one item to another (standard deviation between 1.96 and 2.89). Neither floor nor ceiling effects were observed in the population overall.

### Internal consistency of MAThyS

The Cronbach's alpha coefficient found is 0.95, showing a high consistency.

### External validity

The MathyS total score is moderately correlated of both the MADRS scale (depressive score; r = -0.45) and the MAS scale (manic score; r = 0.56).

### Exploratory psychometric analyses

**Figure 1:**
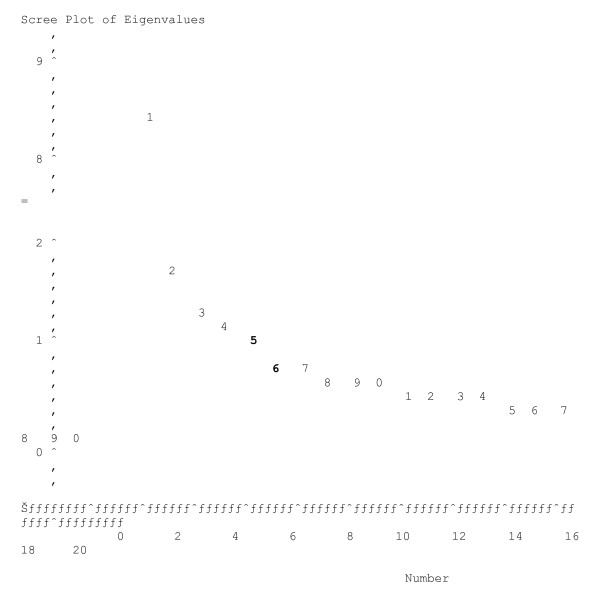
Eigenvalue calculation and diagram (N = 187).

When considering the Kaiser-Guttman rule (17) ("eigenvalues greater than one", see table 3) and the scree plot (see figure 1), our model of 5 factors seems to be valid. The four first factors have an eigenvalue greater than 1.0 and the eigenvalue of the factor five is 0.97. In the scree plot, the "elbow", or the point at which the curve bends, indicates 5 factors to extract. Thus, the number factors to be extracted was fixed at 5, as proposed in the theorical model.

**Table 3:** Eigenvalues of the correlation matrix

	Eigenvalues	Proportion of variance	Cumulative variance
1	8.45	0.42	0.42
2	1.77	0.09	0.51
3	1.35	0.07	0.58
4	1.11	0.06	0.63
5	0.97	0.05	0.68

**Table 4:** MAThyS 5-factor model (Varimax rotation – N = 187). Rotated Factor Pattern

	**Fact 1 ** **EMOTION**	**Fact 2** **MOTIVATION** **and psychomotor** **function**	**Fact 3 ** **Sensory ** **perception**	**Fact 4 ** **Interpersonnal** **communication**	**Fact 5 ** **Cognition**
SE 1			0,60		
MO 2		0,57			
**EM 3**	**0,66**				
VO 4				0,68	
CO 5					0,75
SE 6			0,70		
**EM 7**	**0,79**				
SE 8			*0,54*		
CO 9				*0,50*	*0,52*
**EM 10**	**0,66**				
MO 11		0,69			
CO 12	*0,67*	*0,40*			
SE 13			0,60		
CO 14				0,75	
VO 15		0,71			
VO 16		0,75			
VO 17		0,65			
**EM 18**	**0,86**				
MO 19		*0,53*			*0,61*
SE 20			0,55		

Listwise (component < 0.4 were removed for most clarity)

This 5 factors structure explains 68 per cent of variance (table 3).

Factor 1 (emotional reactivity): all items match with the hypothetised structure of the MAThyS

Factor 2 (motivation and psychomotor function): these two dimensions which were initiallly distinct are now associated in a same factor.

Factor 3 (sensory perception): all items match with the hypothetical structure of the MAThyS

Factor 4 (interpersonal communication): (I am withdrawn/I feel outgoing) and (I feel like communicating with other people less than usual/I feel like communicating with other people more than usual). These two items are associated in one factor and might be called: interpersonal communication.

Factor 5 (cognition): this factor get together only two items but which correspond to the cognitive dimension in the initial model.

Eventually, only three items were not clearly belonging to one factor but could considered in both of them. There are the items 9, 12, and 19. The choice was done as a function of the more clinical relevance. The final struture of the MAThyS is given in table 5.

**Table 5:** Factor structure of the MAThyS

Dimension name	Items numbers	Continuum
EMOTION	3, 7, 10, 18	Hypo-reactivity/Hyper-reactivity
MOTIVATION AND PSYCHOMOTOR FUNCTION	2, 11, 12, 15, 16, 17, 19	Decrease/Increase
SENSORY PERCEPTION	1, 6, 8, 13, 20	Decrease/Increase
INTERPERSONAL COMMUNICATION	4, 14	Decrease/Increase
COGNITION	5, 9	Decrease/Increase

Table 6 gives an overview of the characteristics of the population as a whole and group by group using the new struture of MAThyS. We excluded in this analysis patients with mixed features in order to have means for pure depressive state (supposed to have the lowest scores) and manic states (supposed to have the highest scores). Controls and euthymic bipolar subjects have similar scores close to the theorical mean of 100 (103 for controls, 106 for euthymic bipolar subjects). Depressed patients show low scores in all dimensions while the opposite is observed for manic or hypomanic patients.

**Table 6:** Mean (SD) of the five MAThys dimensions and of the MAThyS total score and sub-scores for each group.

**Mean (SD)**	**Control**	**Euthymic**	**MDE**	**MA**
**N**	44	43	30	31
**Emotion**	21.49 (2.51)	22.96 (4.46)	12.67 (9.72)	31.59 (7.2)
**Motivation**	35.57 (4.95)	35.44 (7.13)	12.4 (6.61)	48.19 (14)
**Sensory perception**	26.14 (2.81)	26.91 (4.32)	18.9 (6.54)	35.95 (9.68)
**Interaction**	10.72 (1.6)	10.55 (3.17)	4.6 (2.94)	14.32 (4.9)
**Cognition**	9.6 (1.9)	10.34 (2.7)	5.8 (3.7)	14.17 (3.7)
**Total**	103.60 (9.77)	106.2 (14.50)	54.42 (22.95)	144.24 (28.39)

Euthymic = Euthymic bipolar patients, MDE = Major Depressive Episode, MA = Manic and Hypomanic Episodes

## Discussion

The descriptive analyses are in support of a good acceptability of the scale. Redundancy between items is low and MAThyS scale indicates moderated and coherent links with the MADRS and MAS scales.

The factor analysis allows to offer a model very close to the initial structure. Two factors correspond exaclty to the items supposed to be in the two corresponding initial dimensions (emotional reactivity and sensory perception). Two previous dimensions, are in fact non independant and are considered now as a single one (motivation and psychomotor function). It is amazing to notice that Kraepelin described these two dimension as a common one. One factor is renamed (interaction) to better fit with the items. The factor 5 defines the cognitive dimension. Concerning the external validity of the new structure of MAThyS, the different groups constituting the studied sample have scores corresponding to their clinical characteristics.

The goal of MAThyS is to provide a total score corresponding clinically to an inhibition (low score)/activation (high score) process. However, it could be useful to describe sub-scores in order to better describe bipolar mood states. Emotional reactivity seems a very useful dimension helping us to distriminate mood states with mixed characteristics [18,19] and has very good psychometric properties.

The scale can also be implemented to obtain a total score enabling comparison of the state of a patient over time. When the subject starts a thymic episode, the MAThyS score will vary according to his/her thymic state, upwards in a state of exaltation and downwards in a state of inhibition. When the episode resolves, the score will tend towards the mean (100).

There are some limitations and advantages for this scale. This scale was not contruct on a classical model because the normal state (corresponding to euthymic state) is between two pathological states. This particular construction is due to the possible fluctuation of the mood in two opposite ways. The advantage of a visual analogic scale is that subjects do not have to make binary decisions, or to refer to a norm. The self-administered questionnaire is assisted (a verbatim and a guidebook are available and can be provided by the corresponding author) in order to help clinician to use the scale. In the future, it could be interesting to developp a clinician rating scale.

Preliminary results using this scale have shown that MAThyS seems appropriate to define a broad mixed state spectrum including a relevant number of patients who would be diagnosed as major depressive episode according to DSM-IV [18]. Moreover, bipolar depressive states are not homogeneous and this dimensional approach is useful for discriminating different forms of bipolar depression. Bipolar depressions may be classified as hypo-reactive or hyper-reactive. This classification might have therapeutic implications because hyper-reactive depression with a moderate global score should belong to a broad spectrum of mixed states [19].

## Conclusion

The characterisation of bipolar mood states based on a global score assessing inhibition/activation process associated with emotional reactivity (rather than the classical opposition euphoria/sadness) can be useful to order thymic states on a continuum and define a spectrum of mixed states. Currently, another proposition is to define this spectrum based on a categorical approach consisting in counting manic and depressive symptoms [4]. A dimensional approach could be more appropriate to understand the mechanisms underlying this spectrum. Further studies are needed to assess if MAThyS may be use as an indicator of the response to treatment.

## Competing interests

The authors declare that they have no competing interests.

## Authors' contributions

CH participated in the design of the study and drafted the manuscript.  KMB participated in the design of the study and included patients.  FM performed the statistical analysis with RP who drafted the statistical section. BF participated in the design of the study and supervised statistical analysis. All authors read and approved the final manuscript.

## Pre-publication history

The pre-publication history for this paper can be accessed here:



## Supplementary Material

Additional file 1 MAThyS (Multidimensional Assessment of Thymic States) by Henry et al. This scale aims **to evaluate your mood **during **the last week**. For each item, indicate how you usually feel by making a vertical line between the two opposite statements. Click here for file
