# Supplementary material for: Construction and validation of a dimensional scale exploring mood disorders: MAThyS (Multidimensional Assessment of Thymic States)
Source: BMC Psychiatry. 2008 Sep 19;8:82. doi: 10.1186/1471-244X-8-82 (PMC2561027; doi:10.1186/1471-244X-8-82)
Supplement: Additional file 1 — MAThyS (Multidimensional Assessment of Thymic States) by Henry et al. This scale aims to evaluate your mood during the last week. For each item, indicate how you usually feel by making a vertical line between the two opposite statements. [file 1471-244X-8-82-S1.doc]

# MAThyS (Multidimensional Assessment of Thymic States) by Henry et al.

This scale aims **to evaluate your mood** during **the last week**. For each item, indicate how you usually feel by making a vertical line between the two opposite statements.

| 1- I am less sensitive to colours than usual |  | I am more sensitive to colours than usual |
| --- | --- | --- |
| 2- I do not feel tense |  | I have a lot of tension |
| 3- I feel emotionally numb |  | I lose control over my emotions sometimes. |
| 4- I am withdrawn |  | I feel outgoing |
| 5- I'm easily distracted and the slightest thing attracts my attention |  | I am not distracted by things going on around me. |
| 6- I am more sensitive to touch than usual |  | I am less sensitive to touch than usual |
| 7- My mood seems to vary a lot, depending on my environment |  | My mood is stable and changes little |
| 8- I am particularly sensitive to music |  | I am less affected by music than usual |
| 9- My mind never stops |  | My mind seems to be functioning in slow motion |
| 10- I am more responsive than usual to things going on around me |  | I am less responsive than usual to things going on around me. |
| 11- I have no energy |  | I feel very energetic. |
| 12- I feel like my thoughts are slowed down |  | I feel like my ideas are racing though my head |
| 13- I find food tasteless |  | I like to eat because I appreciate how food tastes more than usual |
| 14- I feel like communicating with other people less than usual |  | I feel like communicating with other people more than usual |
| 15- I lack the motivation to do new things ? |  | I am coming up with lots of new plans |
| 16- My lack of interest in doing things interferes with managing daily life |  | (I am more interested in doing things.) |
| 17- I am making decisions faster than usual |  | I am finding it harder than usual to make decisions |
| 18- My emotions are very intense. |  | My emotions are not very strong |
| 19- I am moving slowly |  | I feel restless or physically agitated |
| 20- I seem to be less sensitive to smells than usual |  | I seem to be more sensitive to smells than usual |

| **Score:** |  |  |  |
| --- | --- | --- | --- |

Over the course of this period, indicate the types of emotion you have experienced, and how often you have experienced them:

**Sadness**  Never  Occasionally  Often  Very often  Constantly

**Joy**  Never  Occasionally  Often  Very often  Constantly

**Irritability**  Never  Occasionally  Often  Very often  Constantly

**Panic**  Never  Occasionally  Often  Very often  Constantly

**Anxiety**  Never  Occasionally  Often  Very often  Constantly

**Anger**  Never  Occasionally  Often  Very often  Constantly

**Euphoria**  Never  Occasionally  Often  Very often  Constantly

If you have felt other emotions, please specify them below:

**NAME:**

**DATE:**
